# CRISPR/Cas9 Gene Editing System Can Alter Gene Expression and Induce DNA Damage Accumulation

**DOI:** 10.3390/genes14040806

**Published:** 2023-03-27

**Authors:** Lan Yang, Hao Li, Yao Han, Yingjie Song, Mingchen Wei, Mengya Fang, Yansong Sun

**Affiliations:** 1 State Key Laboratory of Pathogen and Biosecurity, Beijing Institute of Microbiology and Epidemiology, Beijing 100017, China; poplarorchid@163.com (L.Y.); lihao88663239@126.com (H.L.); hanyaohyhy@163.com (Y.H.); ylsong72@163.com (Y.S.); weimingchen2021@163.com (M.W.); fangmy2023@163.com (M.F.); 2 Institute of Public Health, Mudanjiang Medical University, Mudanjiang 157000, China; 3 Institute of Veterinary Medicine, Yangzhou University, Yangzhou 225009, China

**Keywords:** CRISPR/Cas9, gene editing, DNA damage, transcriptome

## Abstract

Clustered regularly interspaced short palindromic repeats (CRISPR) and the associated protein (Cas) gene editing can induce P53 activation, large genome fragment deletions, and chromosomal structural variations. Here, gene expression was detected in host cells using transcriptome sequencing following CRISPR/Cas9 gene editing. We found that the gene editing reshaped the gene expression, and the number of differentially expressed genes was correlated with the gene editing efficiency. Moreover, we found that alternative splicing occurred at random sites and that targeting a single site for gene editing may not result in the formation of fusion genes. Further, gene ontology and KEGG enrichment analysis showed that gene editing altered the fundamental biological processes and pathways associated with diseases. Finally, we found that cell growth was not affected; however, the DNA damage response protein—γH2AX—was activated. This study revealed that CRISPR/Cas9 gene editing may induce cancer-related changes and provided basic data for research on the safety risks associated with the use of the CRISPR/Cas9 system.

## 1. Introduction

Clustered regularly interspaced short palindromic repeats (CRISPR) and the associated protein (Cas) systems provide natural adaptive immunity to various bacteria against invading nucleic acids [1,2]. According to the different Cas proteins, the currently known CRISPR/Cas systems are divided into two categories, type 1 and type 2, each containing three types. The type 1 systems are further subdivided into types I, III, and IV, whereas the type 2 systems are subdivided into types II, V, and VI. The type II systems require only one protein component for the interference; thus, they have been used to develop powerful genome engineering technology [3,4,5]. Cas9, an effector protein of the type II CRISPR/Cas system, is the first CRISPR-associated effector protein to be repurposed for genome editing. The CRISPR/Cas9 genome editing system contains the Cas9 protein and single-guide RNA (sgRNA). The Cas9-sgRNA effector complex locates and cuts the target sequences to induce double-stranded DNA breaks (DSBs). Subsequently, the endogenous DNA repair system is activated to repair the DSB and to generate insertions and deletions [5,6,7].

The risks of using exogenous genome-cutting systems gradually emerged as their use became widespread. However, continuously active CRISPR systems also have safety concerns. Researchers have demonstrated that CRISPR/Cas9 could induce large chromosomal deletions on the scale of megabases [8,9]. They also disrupt the nuclear structure, leading to the formation of micronuclei and chromosomal bridges, ultimately resulting in chromosome fragmentation [10]. Moreover, CRISPR/Cas9 gene editing can activate the p53 pathway in multiple cell lines and promote the selective enrichment of p53-inactivating mutations [11,12,13]. Recently, previous studies have found that classical CRISPR/Cas9 technology could cause large-scale DNA rearrangements through reverse transcriptional translocation in multiple human cell lines [14,15]. Aneuploidy and chromosomal truncations occur frequently in human T cells upon CRISPR/Cas9 cleavage using clinical gRNA sequences [16]. Importantly, Wu et al. [17] found that the chromosomal translocations, large-scale loss of chromosomes, and viral DNA insertions caused by gene editing do not disappear with time and continue to remain at high levels, showing a clear random clonal expansion. Höijer et al. [18] found that both the first and second generations of zebrafish had structural variations in the edited fertilized eggs, and these variations were both on- and off-target. However, mitigation strategies for the potential risks involving CRISPR/Cas9 technology remain lacking, and further research is needed to investigate the mechanisms related to CRISPR safety.

In this study, gene expression was characterized by targeting two classical loci, EMX1 and AAVS1, using CRISPR/Cas9. Common differentially expressed genes (DEGs) were selected to reflect the changes associated with the DSBs induced by CRISPR/Cas9. DSBs were found to influence multiple fundamental biological processes and pathways associated with various diseases. This study emphasizes that the additional effects of CRISPR-induced DSBs should be investigated, and provides data to support CRISPR safety research.

## 2. Materials and Methods

### 2.1. Plasmid Construction

The PX459 plasmid encoding Cas9 and sgRNA was purchased from Addgene (Addgene plasmid #48139). sgRNAs (sgRNA_EMX1: 5′-GTCACCTCCAATGACTAGGG-3′; sgRNA_AAVS1: 5′-GGGCCACTAGGGACAGGAT-3′) were cloned into the pX459 plasmid using the *BbsI* restriction sites. The sequencing was performed by Sangon Biotech Co., Ltd. (Beijing, China).

### 2.2. Cell Culture and Transfection

The HEK293 cell line was obtained from American type culture collection (ATCC). The HEK293 cells were maintained in Dulbecco’s modified Eagle’s medium (DMEM, Gibco, Grand Island, NY, USA), supplemented with 10% fetal bovine serum (Excell, Shanghai, China) and 1% penicillin-streptomycin (Gibco, Grand Island, NY, USA), in an incubator under 5% CO_2_ at 37 ℃. Thereafter, the HEK293 cells were seeded in 24-well plates (2 × 10^5^ cells well^−1^) and incubated overnight. Then, the cells were transfected with 500 ng CRISPR plasmids mixed with Opti-MEM (Gibco, Grand Island, NY, USA) and 1 μL Lipofectamine LTX (Invitrogen, Carlsbad, CA, USA) according to the manufacturer’s recommendation. The cells were collected at 24, 48, and 72 h after transfection. Then, the cell samples of each 24-well plate were mixed; half of the cells were used for DNA extraction to detect the gene editing efficiency, and the other half was used for RNA extraction to perform RNA-seq.

### 2.3. Detection of Gene Editing Efficiency via T7EI Assay

The genomic DNA was extracted using the TIANamp Genomic DNA Kit (TIANGEN). The target regions were amplified using Q5 High-Fidelity 2X Master Mix (New England Biolabs, Beverly, MA, USA) and PCR according to the manufacturer’s protocol. The primer sequences used were as follows: EMX1-F: 5′-AACTCGTAGAGTCCCATGTC-3′; EMX1-R: 5′-GAGAAGGCCAAGTGGTCCCA-3′; AAVS1-F: 5′-GCTCTCCCTCCCAGGATCCT-3′; and AAVS1-R: 5′-ACCCCATGCCGTCTTCACTC-3′. The PCR products were denatured at 95 °C for 10 min and reannealed at 25 °C, followed by incubation with 5U T7EI enzyme (New England Biolabs, Beverly, MA, USA) at 37 °C for 30 min. The products were electrophoresed on a 2% agarose gel. Band densitometry analysis was performed using the ImageJ software. The estimated editing efficiency was calculated as previously described [6].

### 2.4. Transcriptome Sequencing and Analysis

The total RNA was isolated from the transfected cells using a TRIzol reagent (Invitrogen, Carlsbad, CA, USA) according to the manufacturer’s instructions. The total RNA amount and integrity were assessed using an Align 2100 system (Agilent Technologies, Santa Clara, CA, USA). RNA-seq libraries were constructed using the Next Ultra™ RNA Library Prep Kit for Illumina (NEB) following the manufacturer’s protocol. The libraries were sequenced by Novogene Co., Ltd., Beijing, China, using the Illumina platform. Raw reads were processed by removing the adaptor reads and low-quality tags. Clean data were aligned with the human genome hg38 using HISAT2 software v2.0.5. The differentially expressed mRNAs with |Log2FC| >  0 and *p* value < 0.05 were selected using DESeq2 1.20.0. Quantitative and differential analyses of alternative splicing (AS) events were performed on the RNA-Seq data using rMATS 4.1.0. The rMATS software can classify AS events into five categories: skipped exon (SE), retained intron (RI), alternative 3′ splice site (A3SS), alternative 5′ splice site (A5SS), and mutually exclusive exon (MXE). The threshold for screening AS events with significant differences was an FDR of <0.05. The STAR-Fusion 1.9.0 software was used to detect the fusion genes. Fusion transcripts were detected using the fusion output results of the STAR alignment. The package was divided into various steps, such as STAR Comparison, STAR-Fusion.predict, and STAR-Fusion. Filter: the verification tool FusionInspector was used to correct the prediction results of STAR-Fusion to ensure the accuracy of the fusion gene results. Gene Ontology (GO) and Kyoto Encyclopedia of Genes and Genomes (KEGG) enrichment analyses of DEGs were performed using NovoMagic tools (https://magic.novogene.com/ (accessed on 1 February 2023). The enrichment results were visualized using http://www.bioinformatics.com.cn (accessed on 6 February 2023), an online platform for data analysis and visualization. Bar plots were constructed using GraphPad Prism 8.

### 2.5. CCK-8 Assay

Cell growth was measured using the Cell Counting Kit-8 (CCK8; Beyotime, Shanghai, China) according to the manufacturer’s instructions. The HEK293 cells were seeded in 96-well plates (1 × 10^4^ cells well^−1^) and incubated overnight. The cells were transfected with 0.15 µg CRISPR plasmids. The CCK-8 was added to each well 48 h after transfection, and the cells were incubated at 37 °C for 1 h. The optical densities (ODs) were measured at 450 nm using a Multiskan FC microplate reader (Thermo Fisher Scientific, Waltham, MA, USA)).

### 2.6. Western Blot Analysis

The transfected cells were lysed using a RIPA buffer (Abcam, Cambridge, MA, USA) and protease inhibitor cocktail (Abcam, Cambridge, MA, USA). The total proteins were separated using 4–12% SurePAGE gel (GenScript, Nanjing, China) and transferred to polyvinylidene difluoride (PVDF) membranes (0.45 µm; Millipore, Billerica, MA, USA), followed by blocking using the blocking buffer (5% skim milk (BD, Franklin Lakes, NJ, USA) in TBST). Thereafter, the membranes were incubated overnight with primary antibodies at 4 °C, followed by incubation with secondary antibodies for 1 h at 25 °C. The membranes were visualized using the ECL substrate kit.

## 3. Results

### 3.1. Experimental Design

**Figure 1:**
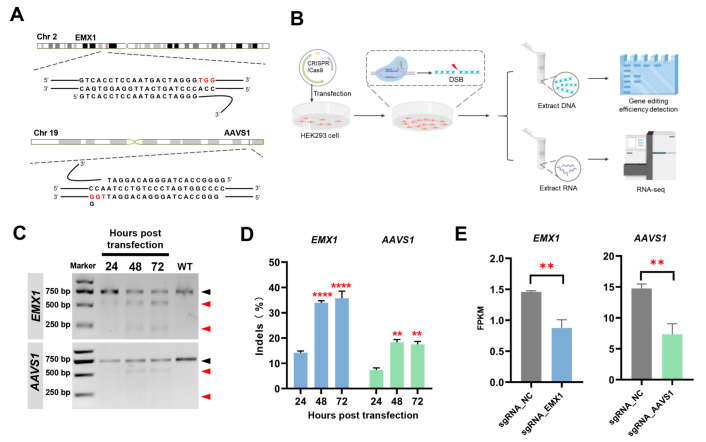
(**A**) The sgRNA sequences of EMX1 and AAVS1 sites. (**B**) The workflow of the present study. The schematic diagrams were generated using Figdraw (https://www.figdraw.com/ (accessed on 19 February 2023). (**C**) Representative agarose gel plot to detect gene editing efficiency by T7E1 assay. (**D**) Statistical results of gene editing efficiencies at EMX1 and AAVS1 sites at 24, 48, and 72 h post-transfection. Biological repeat *n* = 3. Data are presented as the mean ± SEM; *p* values were calculated using unpaired *t*-test; ** *p* < 0.01; **** *p* < 0.0001. (**E**) The gene FPKM of EMX1 and AAVS1 at 48 h post-transfection. Biological repeat *n* = 4. Data are presented as the mean ± SEM; *p* values were calculated using unpaired *t*-test; ** *p* < 0.01. FPKM: Fragments Per Kilobase of exon per Million mapped fragments. sgRNA_EMX1: transfected with a CRISPR/Cas9 system targeting the EMX1; sgRNA_AAVS1: transfected with a CRISPR/Cas9 system targeting the AAVS1; sgRNA_NC: transfected with a CRISPR/Cas9 system that has no targets.

In this study, we selected two target sites, EMX1 and AAVS1, to exclude the influence of the target site function. These are two classic sites for gene editing research and are present on a single locus in the genome, thus providing stable targets for establishing damage models using CRISPR/Cas9. Two sgRNAs were selected according to a previous study [5,6], and the sgRNA sequences were cloned into a CRISPR plasmid as previously described (Figure 1A). The HEK293 cells were transfected with the CRISPR plasmid (Figure 1B) and examined at 24, 48, and 72 h post-transfection. First, we examined the indels in the genomes after gene editing to determine the occurrence of DSBs. Gene editing began at 0–24 h. The editing efficiency at 48 h was significantly higher than that at 24 h, indicating that this was the active period. Only a small amount of gene editing occurred at 48–72 h (Figure 1C,D). To explore the effect of the CRISPR-induced DSBs on the host, the samples at 24 h and 48 h were selected for the RNA-Seq analysis. sgRNA_EMX1 and sgRNA_AAVS1 were the groups of CRISPR/Cas9 targeting the EMX1 and AAVS1 sites, respectively; sgRNA_NC was the negative control group with no targeting sgRNA. The strict negative control eliminated the influence of various factors, such as transfection. The transcriptome analysis validated the decreased expression of *EMX1* and *AAVS1* upon targeting by CRISPR/Cas9 at 48 h post-transfection (Figure 1E).

### 3.2. Transcriptomic Analysis Revealed that Gene Editing Reshapes Gene Expression

**Figure 2:**
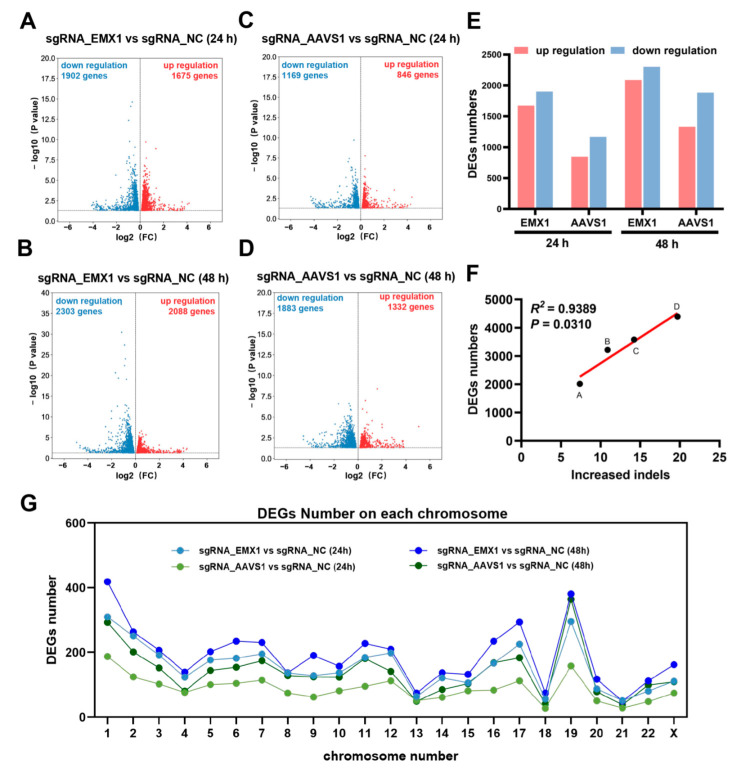
(**A**,**B**) Volcano plots showing the differentially expressed genes (DEGs) between the sgRNA_EMX1 and sgRNA_NC groups at 24 h (**A**) and 48 h (**B**) after transfection. (**C**,**D**) Volcano plots showing the DEGs between the sgRNA_AAVS1 and sgRNA_NC groups at 24 h (**C**) and 48 h (**D**) after transfection. (**E**) Histogram showing DEG distribution at 24 h and 48 h after transfection. (**F**) The correlation between gene editing efficiency and the number of DEGs. (**G**) The distribution of DEGs on each chromosome.

To explore the potential influence of CRISPR gene editing, we examined DEGs in two damage models. The transcriptomic analysis revealed that the gene expression profiles of both the sgRNA_EMX1 and sgRNA_AAVS1 groups were significantly different from those of the sgRNA_NC group. At 24 h post-transfection, the transcriptomic analysis identified 1675 upregulated and 1902 downregulated genes in sgRNA_EMX1 (Figure 2A), and 864 upregulated and 1169 downregulated genes in sgRNA_AAVS1 (Figure 2B). At 48 h post-transfection, there were 2088 upregulated and 2303 downregulated genes in sgRNA_EMX1 (Figure 2C), and 1332 upregulated and 1883 downregulated genes in sgRNA_AAVS1 (Figure 2D). As mentioned above, the change in the mRNA levels of sgRNA_EMX1 was more dramatic than that in the sgRNA_AAVS1 mRNA levels (Figure 2E).

Based on the above observations, both groups showed an increase in the number of DEGs between the two adjacent time points (Figure 2E), which was consistent with an increase in the gene-editing efficiency. Interestingly, the increase in the editing efficiency between the two adjacent time points was linearly related to the DEGs screened in the transcriptome (*R^2^* = 0.9389, *p* = 0.0310; Figure 2F). For point A, at 24 h post-transfection, the average percentage of indels at the AAVS1 site was 7.39%, and the number of DEGs between sgRNA_AAVS1 and sgRNA_NC was 2015. For point B, the difference in the average percentage of indels at the AAVS1 site between 24 h and 48 h post-transfection was 10.89%; the gene editing that occurred during this period triggered changes in the gene expression at 48 h, and the number of DEGs between sgRNA_AAVS1 and sgRNA_NC was 3215 at 48 h post-transfection. For point C, at 24 h post-transfection, the average percentage of indels at the EMX1 site was 14.22%, and the number of DEGs between sgRNA_AAVS1 and sgRNA_NC was 3577. For point D, the difference in the average percentage of indels at the EMX1 site between 24 h and 48 h post-transfection was 19.71%, and that in the number of DEGs between sgRNA_AAVS1 and sgRNA_NC was 4391 at 48 h post-transfection. Overall, the results indicated that gene editing leads to changes in the expression of a large number of genes in the host cell and that the number of DEGs is related to the gene editing efficiency.

Recent studies have reported that Cas9 nuclease induces unwanted chromosomal truncations. Therefore, we explored whether the DEGs were concentrated on the chromosomes with target locus. EMX1 and AAVS1 are located on chromosomes 2 and 19, respectively. The distribution of DEGs on each chromosome was determined after targeting EMX1 and AAVS1 using CRISPR/Cas9, and the distribution of DEGs was found to be similar (Figure 2G). Thus, when CRISPR/Cas9 targets a single site, the CRISPR-induced DEGs are randomly distributed on each chromosome and are not concentrated on the chromosome where the target site is located.

### 3.3. Gene Editing Randomly Induces Alternative Splicing and Little Fusion Genes

**Figure 3:**
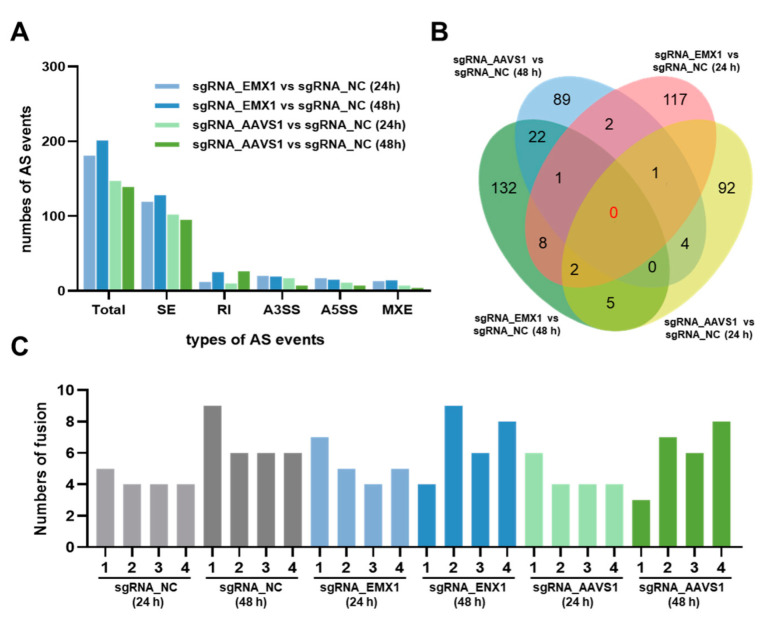
(**A**) The types and numbers of alternative splicing (AS) events in sgRNA_EMX1 vs. sgRNA_NC and sgRNA_AAVS1 vs. sgRNA_NC. (**B**) The common genes associated with AS events between different groups. (**C**) The number of fusion genes in every sample.

Alternative splicing (AS) is an important mechanism that regulates gene expression and protein variability. AS events were predicted using bioinformatic analysis. The results showed that the proportions of the five types of AS events were similar between sgRNA_EMX1 and sgRNA_AAVS1. SE was the most common type of AS event, followed by RI, A3SS, A5SS, and MXE (Figure 3A). However, there were no overlapping genes with AS events among the four sets of DEGs when the sgRNA_EMX1 and sgRNA_AAVS1 cells were compared with the sgRNA_NC cells at 24 h and 48 h (Figure 3B), indicating that the AS events were randomly induced by CRISPR gene editing.

Finally, the fusion genes were identified in every sample. Gene fusions occur through genomic rearrangements and are associated with the occurrence and development of various cancers. The results revealed a small number of fusion genes (Figure 3C), which might have formed randomly as the cells grew, independent of the CRISPR gene editing process. Thus, we speculated that low-damage DSBs at a single site may not be sufficient to trigger genome rearrangements, resulting in fusion genes.

### 3.4. Gene Editing Altered Multiple Fundamental Biological Processes and Pathways Associated with Cancer and Other Diseases

**Figure 4:**
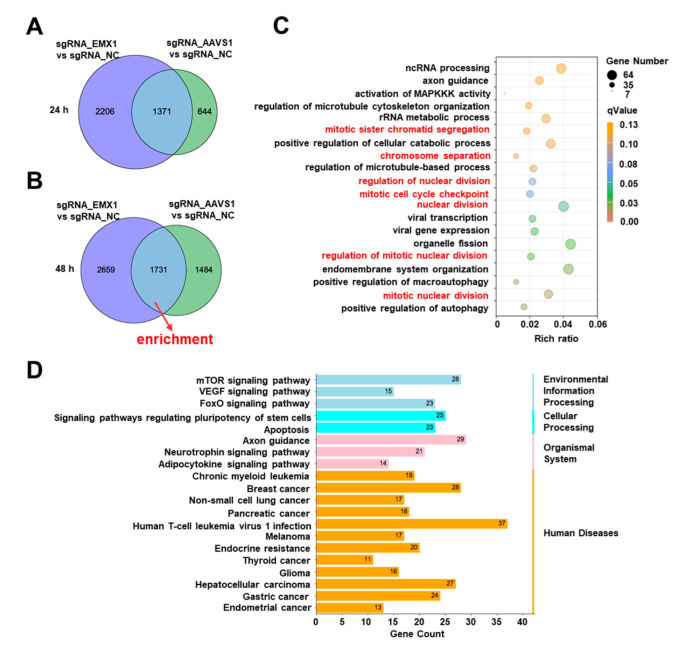
(**A**,**B**) Venn diagram of DEGs with the common change between sgRNA_EMX1 vs. sgRNA_NC and sgRNA_AAVS1 vs. sgRNA_NC at 24 h (**A**) and 48 h (**B**) after transfection. The middle circle indicates the common DEGs between the two groups. (**C**) GO enrichment analysis of the common DEGs, the red color represents the pathways related to chromosome segregation. Rich ratio: the ratio of the number of DEGs annotated to the GO number to the total number of DEGs. qValue: *p*-value after multiple hypothesis testing. (**D**) KEGG analysis of the common DEGs.

There were many overlaps in the DEGs between sgRNA_EMX1 and sgRNA_AAVS1, compared with that in the sgRNA_NC cells. A total of 1371 genes at 24 h (Figure 4A) and 1731 genes at 48 h were shared between the two groups (Figure 4B). These common genes may be responsible for the gene-editing process, which excludes site-specific interference. Common DEGs at 48 h were selected for functional enrichment analysis. To describe the functions and pathways of common DEGs, we performed GO and KEGG enrichment analyses.

Notably, several fundamental cellular processes were altered due to gene editing. The significantly enriched biological processes were gene expression including ncRNA processing, rRNA metabolic processes, viral gene expression, and viral transcription; chromosomal regulation including mitotic sister chromatid segregation and chromosomal separation; and cell division including the regulation of nuclear division, organelle fission, and mitotic cell cycle checkpoint (Figure 4C). Interestingly, the GO enrichment analysis revealed the activation of viral- and immune-related biological processes, including viral gene expression, viral transcription, the positive regulation of macroautophagy, and the positive regulation of autophagy. The results indicated that the CRISPR gene editing process changes the fundamental biological processes of cells and can cause a major crisis.

To inspect the pathways identified by the CRISPR gene editing in greater detail, we performed a KEGG enrichment analysis based on the common DEGs. The enrichment results were focused on human disease-related pathways, including breast cancer, non-small cell lung cancer, pancreatic cancer, mammalian target of rapamycin (mTOR), vascular endothelial growth factor (VEGF), and forkhead box proteins of the class O subgroup (FoxO) signaling pathway (Figure 4D). These results suggest that gene editing reshapes the transcriptome, causing cells to undergo carcinogenesis-like changes, highlighting potential oncogenic risks. Therefore, when conducting scientific research we should be focused on the effects of DSBs induced by CRISPR/Cas9.

### 3.5. CRISPR Gene Editing Activates DNA Damage Markers γH2AX

**Figure 5:**
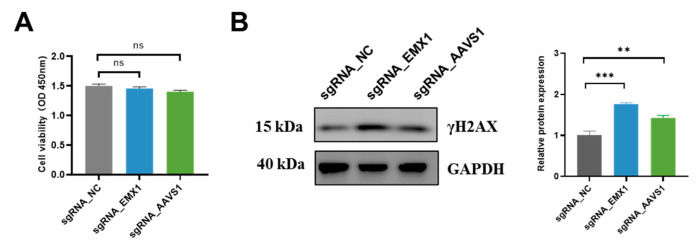
(**A**) The cell activity after gene editing. Biological repeat *n* = 2. Data are presented as the mean ± SEM; *p* values were calculated using unpaired *t*-test; ns, no significance. (**B**) The relative expression levels of γH2AX. Biological repeat *n* = 3. Data are presented as the mean ± SEM; *p* values were calculated using unpaired *t*-test; ** *p* < 0.01; *** *p* < 0.001.

Abnormalities in DNA damage and repair are considered one of the causes of numerous diseases. Therefore, we examined the effects of CRISPR gene editing on cell growth and DNA damage. At 48 h after CRISPR transfection, the cell growth in the sgRNA_EMX1 and sgRNA_AAVS1 groups was not different from that in the sgRNA_NC group, indicating that the cell growth was not affected (Figure 5A). However, the DNA damage marker γH2AX was detected, and the results showed that targeting two single sites, even with low editing efficiency, also triggers the accumulation of extensive DNA damage, which may trigger the aforementioned changes in the cell division processes and cancer pathways (Figure 5B).

## 4. Discussion

Gene editing using CRISPR/Cas9 induces DSBs and various other catastrophic events, such as genomic rearrangements and chromosomal variations [10,17,18]. Clinical trials involving CRISPR-Cas9 have emphasized that safety is a limitation of its applications [19,20,21]. In this study, we investigated the potential impact of CRISPR/Cas9 gene editing on DNA damage. This study revealed safety concerns regarding the application of CRISPR/Cas9 gene editing and suggested the targets that should be focused on.

First, we established a low-damage model using EMX1 and AAVS1 sgRNAs; the indels were <50%. The gene expression was reshaped, as previously found in CRISPR-induced broad damage models [22]. The changes in the mRNA levels were positively correlated with the gene editing efficiency. These DEGs were randomly distributed on each chromosome. Additionally, AS events were found to play a key role in the transmission of genetic information and increase the adaptability of cells to thrive in different environments. Zhang et al. [23] accidentally found several alternatively spliced mRNAs upon using the CRISPR/Cas9 system for gene knockout. Some mRNAs have been shown to skip entire exons or use new donors and accept splice sites, resulting in consecutive exon deletions The results of this study showed that the CRISPR-induced AS events were random, and there was no similarity in the location of the AS events at different sites. Therefore, attention should be paid to AS events when performing gene editing using CRISPR/Cas9 that can generate novel proteins, some of which may be highly oncogenic. Moreover, CRISPR has been reported to trigger changes in the chromosomal structure [10,17]. A fusion gene is a chimeric gene in which all or part of the sequences of two genes are fused, generally caused by chromosomal translocation or deletion. In this study, low damage at a single locus did not seem to be sufficient to trigger widespread gene fusions.

Common DEGs between the two targeted sites indicated the presence of DSB-related DEGs. GO and KEGG enrichment analyses were performed. The GO enrichment analysis revealed that the chromosomal segregation and nuclear division were greatly changed during the active period of gene editing. These findings suggest a potential mechanism for the recently observed chromosomal aberrations following gene editing. Zhang et al. [24] discovered that large deletions and translocations occur at average rates of 3.2% and 6.2%, respectively, upon the induction of indels by CRISPR/Cas9. Furthermore, Leibowitz et al. [10] reported that CRISPR/Cas9 gene editing may generate micronuclei and chromosomal bridges and initiate chromothripsis. It is speculated that fundamental biological disorders may be the origin of extensive genetic alterations. Chromosomal fragmentation is closely associated with human congenital diseases and cancers. Similarly, KEGG enrichment revealed that CRISPR-induced DSBs led to the aberrant expression of many cancer- and disease-related pathways. A lower editing efficiency at a single site triggers similar damaging effects. Several studies have reported that CRISPR may trigger changes in the p53 signaling pathway and that the CRISPR gene editing process may induce cancer [11,12,13,25]. The KEGG enrichment analysis showed that several disease-associated pathways were activated. Simultaneously, several tumor-associated signaling pathways were altered. The mTOR signaling pathway is often activated in tumors, which not only regulates gene transcription, protein synthesis, cell proliferation, and immune cell differentiation, but also plays an important role in tumor metabolism. The mTOR protein regulates cell proliferation, apoptosis, and autophagy, and mTOR is consistently stimulated in tumors to maintain tumor cell growth, survival, and proliferation [26,27]. Moreover, VEGF-mediated signal transduction occurs in tumors and their microenvironments. It is beneficial for the development of tumors as it promotes angiogenesis in tumor tissues, the rapid proliferation of tumor tissues, and the proliferation and metastasis of tumor cells [28]. The FoxO signaling pathway plays an important regulatory role in tumor suppression, cell death, and stress regulation. The FoxO protein is involved in the regulation of many processes, such as cell proliferation, apoptosis, and differentiation [29]. The activation of these signaling pathways further suggests that CRISPR may trigger cancer-related changes.

Finally, we found that CRISPR-triggered DSBs did not affect cell growth when targeted at a single locus. Liu et al. [22] reported that targeting repeat sequences using CRISPR can decrease the cell viability. Moreover, Ihry et al. [12] reported massive cell death induced by the gene editing of stem cells. In this study, gene editing at a single site did not cause severe cytotoxicity in HEK293 cells. However, CRISPR-induced DSBs are not immediately repaired by the cells. Western blot analysis revealed that even at a single site, gene editing triggered the increased expression of the damage marker γH2AX.

Taken together, this study revealed that CRISPR-mediated damage models can elicit significant damage and cancer-related risks. This study re-emphasizes the safety concerns regarding CRISPR. In addition, the effect of CRISPR-induced DSB on cells should be noted when using the CRISPR system as a gene editing tool.

## Data Availability

Data is contained within the article. The data presented in this study are available on request from the corresponding author and first author.
